# Arthropod Pest Management in Strawberry

**DOI:** 10.3390/insects13050475

**Published:** 2022-05-19

**Authors:** Sriyanka Lahiri, Hugh A. Smith, Midhula Gireesh, Gagandeep Kaur, Joseph D. Montemayor

**Affiliations:** Department of Entomology, Gulf Coast Research and Education Center, University of Florida, Wimauma, FL 33598, USA; hughasmith@ufl.edu (H.A.S.); mgireesh@ufl.edu (M.G.); gkaur2@ufl.edu (G.K.); montemayorj@ufl.edu (J.D.M.)

**Keywords:** thrips, mites, lygus, spotted wing drosophila, sap beetles, aphids, armyworms, whitefly, integrated pest management, biological control

## Abstract

**Simple Summary:**

Strawberry is a commercially important crop which is produced and consumed globally. As there is an increase in economic significance of strawberry production, growers across the globe face challenges in protecting the crop against insect and mite pests. The damage from insect pests results in significant yield loss which adversely affects the strawberry industry. To overcome this situation, management of pests is warranted with reduced impact on the environment and beneficial organisms. Even though insecticide-driven management practices predominate in the strawberry production system, the use of non-chemical alternatives is also gaining importance. The current review is aimed at discussing the important pests of strawberry and various integrated pest management practices adopted worldwide to reduce the damage impact and improve production.

**Abstract:**

The strawberry crop endures economic losses due to feeding injury from a number of phytophagous arthropod pests. A number of invasive pests have posed challenges to crop protection techniques in the strawberry cropping system recently. It is increasingly evident that sole reliance on chemical control options is not sustainable. In this review, current challenges and advances in integrated pest management of various strawberry pests are presented. Key pests discussed include thrips, mites, lygus bug, spotted wing drosophila, seed bug, weevils, aphids, whiteflies, and armyworms. Several integrated pest management techniques that include use of intercropping, resistant cultivars, irradiation with gamma rays, use of spectral sensitivity of pests, biological control agents and natural enemies, and biorational pesticides have recently been reported to be useful in managing the various strawberry pests. With the increase in world production of strawberry, several techniques will be necessary to manage the pest complex of strawberry.

## 1. Introduction

The strawberry, *Fragaria* × *ananassa* Duchesne (Rosales: Rosaceae) management practices presented in this review focus mostly on the integrated pest management techniques that have been utilized globally within the last 10 years to tackle strawberry pest issues. In recent years, development of resistance to several plant protection products used for managing arthropod pests in strawberries has been reported [1]. In addition to pesticide resistance development, the incidence of strawberry pests expanding their invasive range has increased [2,3,4,5,6]. Strawberry remains a high-value crop worldwide and the number of countries producing this crop is on the rise, as is evident from the increase in its global production of 7.6 million kg in 2014 to 8.9 million kg in 2019 [7]. Demand for organic strawberry is also on the rise, which in turn has increased the requirement for information regarding alternative pest management options. Growers that succeed in producing strawberry sustainably in spite of global competitive markets are poised to generate substantial revenue for their regional and national economies. However, there has been a gap in knowledge about the latest developments worldwide with regards to review articles covering recent strawberry arthropod pest management. Upon searching for review articles on Web of Science^TM^ and Google Scholar, several articles were found that covered management tactics but had the following issues: (1) covered only a single pest; (2) were older than 10 years; (3) were geographically limited to Northern and Central Europe or the United States. Therefore, the aim of this review article is to present alternative management tactics for major arthropod pests of strawberry occurring globally, with potential for implementation in strawberry pest management.

## 2. Strawberry Pests and Their Management

### 2.1. Thrips

Thrips (Thysanoptera: Thripidae) form a complex of species that routinely infest strawberry, at both vegetative and reproductive stages. Pest thrips species cause damage by feeding on leaves, flowers, or fruits, resulting in reduced crop yield and quality. Particularly in strawberry, thrips feeding leads to bronzing, cracking, and cat-facing injuries on the fruits [5,8]. Although thrips-vectored tospovirus and non-viral diseases are common in other cropping systems, such instances of transmission have not yet been reported in strawberry [9]. Even though only 1% of all the thrips species are considered to be serious pests, they cause immense damage to strawberry production worldwide [9,10,11]. The major thrips species that are pests of strawberry include *Frankliniella intonsa* (Trybom), *Thrips tabaci* Lindeman, *Frankliniella occidentalis* Pergande, *Frankliniella bispinosa* Morgan, *Thrips palmi* Karny, *Frankliniella schultzei* Trybom, *Thrips fuscipennis* Haliday, *T. imaginis* Bagnall, *T. atratus* Haliday, and *Thrips major* Uzel [8,12,13,14,15]. *Scirtothrips dorsalis* Hood is an invasive pest in the southeastern USA, which became established in this region on strawberry crops in 2015 [5]. Within a few years of invasion in the USA, *S. dorsalis* has rapidly spread across strawberry and horticultural crop-producing regions of the country [16]. 

Thrips such as *S. dorsalis* or *F. occidentalis* are notoriously difficult to manage due to their small size (1–2 mm) and high reproductive capacity. Moreover, their ability to hide in small spaces within plants also makes management challenging [17]. In addition, there is a high probability of movement of thrips to new geographic areas through the trade of plant materials such as cuttings, seedlings, potted plants, and fresh fruits. Continuous monitoring of thrips pests in the field is essential to implement timely management strategies. Biocontrol agents such as *Neoseiulus cucumeris* Oudemans, *Stratiolaelaps scimitus* (Womersley), *Amblyseius swirskii* Athias-Henriot (Arachnida: Phytoseiidae), and *Orius* spp. are found to be effective in thrips management along with adequate insecticide spray treatments [18,19,20]. Additionally, foliar-dwelling entomopathogenic nematodes, *Thripinema* spp. (Tylenchida: Allantonematidae) are also effective in controlling thrips population [18]. Cultural control methods include eliminating the alternative host or weeds in and around the main crop which can serve as reservoirs for thrips [16]. 

Thrips management in strawberry is mainly insecticide-driven. Conventional insecticides such as spinetoram, cyantraniliprole, flupyradifurone, imidacloprid + lambda cyhalothrin, and bifenthrin are found to be effective in managing thrips population [10,21]. Research findings suggest that three consecutive applications of neem extract reduced the thrips population by more than 70% in strawberry, which makes it an alternative for the reduced use of synthetic pesticide [22]. However, repeated application of insecticides induces insecticide resistance in thrips, especially with *F. occidentalis* developing resistance to organophosphate, carbamate, and pyrethroid insecticides [23,24]. As a result, it is critical to apply these insecticides in rotation to slow down resistance development in thrips. Additionally, over usage of synthetic pesticides can also affect the beneficial insects. Hence it is important to incorporate an integrated pest management approach for thrips which includes cultural and biological control methods with adequate spray treatments. 

### 2.2. Mites

*Tetranychus urticae* Koch, *Tetranychus cinnabarinus* Boisduval, *Phytonemus pallidus* Banks, and *Polyphagotarsonemus latus* Banks (Trombidiformes: Tarsonemidae) are common mite pests in strawberry, with *T. urticae* being the most consistently occurring pest of open-field and high tunnel strawberry [1,25,26,27]. With a round and soft body, *T. urticae* have a body length of 350 to 1000 µm and are sexually dimorphic, where males can be distinguished from females by the conical tapering of the caudal end and the larger size of females [28]. *Tetranychus urticae* have two large dark areas on their abdomen and have variable body color including red, orange, green, and yellow [26]. These *T. urticae* adults and nymphs feed on plant cell chloroplasts and typically develop on the adaxial side of leaves where they can form complex webbing. Feeding causes pale chlorotic spots, known as stippling, to appear on the leaves due to the depletion of mesophyll cells, resulting in the breakdown of plant tissue [28,29]. *Tetranychus urticae* uses its stylet to secret enzymes from its salivary gland to aid in the breaking down of plant cells [30]. Additionally, *T. urticae* are capable of arrhenotokous parthenogenesis which means that a virgin female can lay unfertilized eggs that will hatch into males when mature males are unavailable initially during host colonization. Therefore, a single unfertilized female can quickly establish a bisexual population on the host plant [31].

Monitoring and sampling of *T. urticae* is the key to its management. Several management options for *T. urticae* exist for strawberry growers. Steam treatments of transplants can be an effective way of reducing mite populations in the early phase of transplanting. Temperatures of 48 °C have been shown to completely kill adult females after 2.7 h of exposure and eggs after 1.9 h of exposure [32]. Several predatory mites belonging to the family Phytoseiidae are sold commercially as biological control agents [33]. A commonly used mite predator, *Phytoseiulus persimilis* Athias-Henriot, is a spider mite specialist. It is known to cause a swift decline of *T. urticae* populations after application. If miticides are applied in conjunction with mite predators, it is recommended that growers wait for 7–10 days before releasing the phytoseiid mites onto strawberry plants [26].

Management options using pesticides are available for both conventionally [34] and organically grown strawberry. Organic products containing azadirachtin, paraffinic oil, *Metarhizium anisopliae* strain F52, potassium salts of fatty acids, *Isaria fumosoroseus* Apopka strain 97, unsulfonated residue of petroleum oil, neem oil, heat-killed *Burkholderia rinojensis* strain A396, and heat-killed *Chromobacterium subtsugae* strain PRAA4-1 and spent fermentation media are viable options for management [35,36].

*P. pallidus* is a smaller, clear-bodied mite that is ~250 µm in body length. Their eggs are also clear, oval, and have a smooth surface, and are typically glued to the edge of the leaf trichomes or trichomes of the fruit calyx, typically on the underside. Unlike *T. urticae*, these mites are not visible without magnification. *Phytonemus pallidus* prefer to feed on young foliage and are typically found on strawberry trifoliates that are still folded and developing near the crown [37]. Feeding causes the plant to have a stunted appearance (compact leaf mass) until the plant eventually turns brown and dies [26]. Flowers can be damaged at high pest levels, resulting in browning and drying. Fruits may also be affected by feeding damage and will appear undersized. High densities may prevent fruit from being produced entirely [28,37]. Adults can overwinter in the crown of strawberry but in warmer climates they can continue to reproduce through the winter and females are capable of parthenogenesis [38,39].

Research findings suggest that similar control methods for *T. urticae* likely affect *P. pallidus*. Research conducted using hot water treatments found that soaking *P.-pallidus*-infested strawberry runners at 46 °C for 6.5 min killed *P. pallidus* [40]. Moreover, commercially available generalist phytoseiid mites such as *Neoseiulus californicus* McGregor and *Neoseiulus cucumeris* Oudemans have been shown to control phytophagous mites on strawberry [28,39]. For the management of *P. pallidus* in strawberry, prevention of pest infestation and pest establishment is a better tactic than crop rescue, owing to limited miticide compounds available and lack of effective pest suppression. Many miticides labeled for *T. urticae* are not labeled for *P. pallidus* management in strawberry; therefore, manufacturer labels of miticides should be thoroughly referenced before being included in chemical control programs.

### 2.3. Tarnished Plant Bug

The tarnished plant bug, *Lygus lineolaris* (Palisot de Beauvois) (Hemiptera: Miridae) is one of the most damaging pests of strawberry [41]. *Lygus hesperus* Knight is an economically important pest of strawberry on California’s central coast, whereas *Lygus rugulipennis* Poppius affects the late season strawberry production in the UK. *Lygus* spp. has a very broad host range, attacking over 25 economic and 117 weed hosts [42]. Strawberry is not a preferred host; however, *Lygus* spp. migrate from unmanaged areas during the late spring as seasonal rains diminish and establish themselves in strawberries and other crops [43]. *Lygus* spp. have piercing-sucking mouthparts and cause economic damage to strawberry primarily by feeding on the achenes, or seeds of developing fruit [44]. This feeding causes malformation of the fruit, resulting in symptoms described as “cat-facing”. There is evidence that oviposition by *Lygus* spp. into the ripening fruit can also cause cat-facing [43]. Both adult and nymphal *Lygus* spp. feed on strawberry and contribute to economic damage.

Adults are about 6 mm long and are a green-brown color with a reddish tint on the forewings. They have a characteristic triangle in the center of the back that is pale green. Young nymphs are pale green. Older nymphs develop five black spots on the back: four arranged between the wing buds and one in the middle of the abdomen. Eggs are oviposited into plant tissue. Methods for sampling *Lygus* spp. in strawberry have been developed [45,46]. Hand-held vacuum devices and beating trays such as a 12-inch (30 cm) embroidery hoop can be used to sample *Lygus* spp. [47]. In addition, degree day models can be used to predict the establishment of *Lygus* spp. populations in strawberry [48].

*Lygus lineolaris* is primarily controlled in conventional strawberry production with broad spectrum insecticides, including pyrethroid, organophosphate, and neonicotinoid insecticides [49,50]. In addition, newer modes of action including a butenolide (flupyradifurone), a growth regulator (novaluron), and a pyridinecarboxamide (flonicamid) are registered for use against *Lygus* spp. on strawberry. Entomopathogenic fungus, *Beauveria bassiana* (Balsamo-Crivelli) Vuillemin can also contribute to *Lygus* spp. management [51]. An effective push-pull strategy has been described for *Lygus rugulipennis* management in strawberry using synthetic semiochemicals [52]. The authors found hexyl butyrate (HB) to be an effective push element and *L. rugulipennis* female sex pheromone combined with phenylacetaldehyde was used as the pull element. 

Intensive insecticide use to manage *Lygus* spp. is disruptive to biological control programs directed at the management of twospotted spider mites and contributes to the development of populations of *Lygus* spp. that are resistant to insecticides [51]. Augmentative releases of the native egg parasitoid, *Anaphes iole* Girault (Hymenoptera: Mymaridae) and *Leiophron uniformis* (Gahan) (Hymenoptera: Braconidae) have been evaluated for management of *Lygus* spp. [53]. The authors found that *A. iole* has potential as an inundative biocontrol release agent in strawberry owing to high (~80%) parasitism of *L. hesperus* at high release rates. However, the potential of *L. uniformis* as a biocontrol agent needs further investigation as the parasitism of *L. hesperus* also reached 97% at high release rates (100 parasitoids/150 nymphs in cage), but the *L. uniformis* oviposition rate declined with an increase in parasitoid density, unlike in the case of *A. iole*. *Lygus* spp. have been observed to oviposit proportionally more eggs into the fruit (46.5% of eggs laid) than in other parts of the plant, but in young fruits where inter-achene distance was the minimum, parasitism by *A. iole* was lower (25.4%) than in more developed fruit [43]. The authors concluded that the fruit offered a refuge for *Lygus* spp. eggs from parasitism by *A. iole,* necessitating alternate strategies for management. 

Tractor-mounted suction machines, or bug vacs, have been evaluated alone and in combination with insecticide applications for management of *Lygus* spp. [54,55,56]. A clear advantage of integrating bug vacs with insecticidal control has not been demonstrated over sole reliance on insecticides, and in some cases, bug vacs can damage the strawberry crop. Moreover, the use of trap crops in combination with insecticide sprays or physical control methods can increase the efficiency in *Lygus* spp. management. The studies in [41,57] found that vacuuming *Lygus* spp. from the trap crop reduced damage on adjacent strawberry more than directly vacuuming the crop. In addition, trap crops such as alfalfa (*Medicago sativa* L.), can serve as a refuge for natural enemies [58,59].

### 2.4. Beetles

Sap beetles: Although commonly referred to as sap beetles, this strawberry pest consortium is made up of several genera and species. Based on research findings, *Haptoncus luteolus* (Erichson), *Lobiopa insularis* (Castelnau)*,* and *Carpophilus fumatus* Boheman (Coleoptera: Nitidulidae) are considered to be the major species of sap beetles associated with strawberry fruit damage [60,61]. 

The adults are attracted to and feed on ripening fruit, followed by egg laying by the females in the soil surrounding the fruit [62]. The sap beetle larvae emerge from eggs in the soil and feed on the fruits until pupating in the soil. When presented with green, semi-ripe, and ripe strawberry, *L. insularis* clearly show preference for semi-ripe and ripe fruits [63]. The sap beetle *L. insularis* is a polyphagous species that is associated with significant economic losses (20–70%) due to strawberry fruit damage in the USA, Brazil, and Argentina [60,64,65,66]. In addition to causing direct feeding damage by burrowing into the semi-ripe and ripe strawberry, these sap beetles facilitate secondary pest infestations of fruit flies, *Drosophila* spp. (Diptera: Drosophilidae) and phytopathogens such as fungi, *Rhizopus stolonifer* (Mucoraceae) [63].

Although pyrethroids such as bifenthrin and fenpropathrin are labeled for use in strawberry, the best management practice for sap beetle suppression is maintaining field sanitation by removing ripe fruits promptly from fields [62]. Since the larvae hatching from eggs in the soil tend to infest the fruits closest to the soil, strawberry grown on raised beds with fruits never hanging close to the soil surface will remain least affected by sap beetle infestation and population establishment. In fact, strawberry cultivars that hold ripe fruit off the ground were found to sustain significantly less damage from sap beetles when compared to cultivars that have ripe fruit touching the ground [63]. Moreover, limiting the use of fungicides by following an environmental-model-based spray program approach can contribute to 55% reduction in *Stelidota geminate* (Say) infestation in open-field strawberry [67].

Weevils: The black vine weevil, *Otiorhynchus sulcatus* F. (Coleoptera: Curculionidae) was reported as an invasive pest of strawberry in Finland and human activities were mainly held responsible for aiding the spread of this wingless beetle there [2]. Typical of root-feeding weevil pests, the larvae of *O. sulcatus* reduce plant growth by feeding on the strawberry roots, which in acute cases may lead to plant wilting and death [68]. In the UK, neither chemical nor biological control tactics suppressed this pest but host plant resistance in one cultivar “Symphony” showed lower feeding and oviposition by the adult *O. sulcatus* feeding on the leaves of this cultivar [68]. The root weevil species complex affecting strawberry includes *O. sulcatus*, strawberry root weevil, *O. ovatus* L., clay-colored weevil, *O. singularis* L., cribrate weevil, *Otiorhynchus cribricollis* Gyllenhal, Fuller’s rose beetle, *Pantomorus cervinus* Boheman, Woods weevil, *Nemocestes incomptus* Horn, *Barypeithes pellucidus* Boheman, and obscure root weevil, *Sciopithes obscurus* L. [69,70]. Cultural management of root weevils includes keeping strawberry beds free of weeds and grass. Additionally, pesticide spray or dust which contains pyrethroids acts as an effective chemical control option. For biological control, use of entomopathogenic nematode, *Steinernema kraussei* (Steiner, 1923), is found effective [71]. 

The strawberry blossom weevil, *Anthonomus rubi* Herbst (Coleoptera: Curculionidae) is an economically important pest of strawberry. In addition to strawberry plants, *A. rubi* tend to feed and reproduce on raspberries and rarely on blackberries [72]. *A. rubi* is considered to be a key pest of strawberry in the UK and throughout Europe, Asia, and North Africa [73,74]. Additionally, recent reports document that *A. rubi* has been established in the Lower Mainland region of British Columbia, Canada [74]. Adults of *A. rubi* are relatively smaller, ranging from 2.5 to 3.0 mm in length. Female *A. rubi* oviposit eggs singly inside the developing flower buds. Soon after oviposition, the females cut off the bud stalk to prevent bud development and facilitate larval development [75]. The feeding activity of adult *A. rubi* results in small deep holes on berries at different stages, causing significant yield losses. 

Research findings suggest that yellow sticky traps combined with synthetic attractants are efficient for adult monitoring and mass trapping of *A. rubi* [76]. For management, periodic removal of severed buds or adults is found to be effective [76]. Parasitoid wasps from the families Braconidae (Ichneumonoidea) and Pteromalidae (Chalcidoidea) have been recorded as parasitoids of the larval stages of *A. rubi* in Europe [74,77]. However, more studies are warranted to understand the efficacy of these natural enemies as potential biocontrol agents. 

Scarab beetles: In perennial strawberry fields, other root-feeding beetle species include *Popillia japonica* Newman, *Anomala orientalis* Waterhouse, *Amphimallon majalis* Razoumowsky, and *Maladera castanea* Arrow (Coleoptera: Scarabaeidae) [71,72]. Their management options include crop rotation with “Saia” oats, *Avena strigosa* Schreb and “Garry” oats, *Avena sativa* L., and applying neem oil or insecticidal soaps [71,78].

### 2.5. Spotted Wing Drosophila

*Drosophila suzukii* Matsumura (Diptera: Drosophilidae), a native pest of Asia, has invaded fruit crops in Europe and USA and infests unripe strawberry fruits by laying eggs inside the fresh fruit before harvest, using their serrated ovipositor [79]. It has now been reported from the strawberry production regions of Brazil and Africa also [3,80]. This species is particularly devastating as it completes several generations during a field season and is capable of causing farmgate value losses of more than USD 500 M [81]. Resistance to insecticides is an ever-present threat; therefore, alternative pest management tactics are being investigated. It was found that the fruit fly, *Drosophila melanogaster* Meigen, a related species, shows aversion to UV light during oviposition [82]. Inspired by this finding, studies were conducted to understand the spectral sensitivity of *D. suzukii*. It was concluded that there is a preference in females for near-UV range (340, 365, 405 nm) but avoidance of the blue-green light spectrum (430–565 nm) [83]. This study conducted using strawberry fruit shows potential in utilizing the preferential phototactic response to ultra-violet light versus visible light in *D. suzukii*. Another valuable development comes from the investigation of 107 strawberry cultivars, where the authors reported three cultivars that had significantly reduced adult *D. suzukii* emergence until 17 days post-exposure [84]. Additionally, efficacy of multiple natural enemies on immature *D. suzukii* has been studied in both the strawberry and blueberry system. In strawberries, *Orius insidiosus* (Say) (Hemiptera: Anthocoridae) along with *Heterorhabditis bacteriophora* Poinar (Rhabditida: Heterorhabditidae) resulted in 81% reduction of *D. suzukii* [85]. 

### 2.6. Aphids and Whiteflies

Several pests infest strawberry depending on the pest management program, proximity to other host crops, and field sanitation situation. Aphids (Hemiptera: Aphididae) and whiteflies (Hemiptera: Aleyrodidae) infesting strawberry fall in this category and cause yield loss due to feeding on strawberry leaves. Feeding leads to reduction of plant vigor and vector plant viral diseases (strawberry decline disease) and limits the productivity of the crop by production of honeydew which leads to sooty mold [86,87,88]. Several aphid species infest cultivated strawberry around the world including *Aphis ruborum* (Börner and Schilder), *Chaetosiphon fragaefolii* Cockerell, *Aphis forbesi* Weed, *Aphis gossypii* Glover, *Myzus persicae* Sulzer, and *Macrosiphum euphorbiae* Thomas. Whitefly species infesting strawberries include *Trialeurodes vaporariorum* Westwood, *Aleyrodes spiraeoides* Quaintance, and *Bemisia tabaci* Gennadius [51,87].

Aphid parasitoids such as *Aphelinus varipes* (Foerster) (Hymenoptera: Aphelinidae) and *Aphidius colemani* Viereck (Hymenoptera: Braconidae) have been found to be associated with aphid pests and are viewed as important tools in the integrated pest management of aphids in strawberry [88]. Several predators such as syrphid flies (Diptera: Syrphidae), green lacewings (Neuroptera: Chrysopidae), and lady beetles (Coleoptera: Coccinelidae) are effective in suppressing aphid and whitefly populations. Irradiation of boxed fresh fruits with gamma rays (100 Gy) has also shown efficacy in management of *B. tabaci* and *T. vaporariorum* [89]. Additionally, biotechnological traps based on an entomopathogenic fungus, *Isaria fumosorosea* (Wize) Brown and Smith, have been found to be effective in controlling *B. tabaci* adults in strawberry [90].

### 2.7. Seed Bug

Seed bug, *Neopamera bilobata* Say (Hemiptera: Rhyparochromidae) is native to North America and has emerged as an occasional pest of strawberry in USA, Mexico, and Brazil, as they feed on the achenes of green or mature fruits, leading to fruit injury commonly described as “cat-facing” or drying and brown staining of strawberry [91,92,93,94]. Both adults and nymphs are actively feeding stages; these injuries make the fruits unmarketable, especially since adults feed on ripe fruits only whereas nymphs feed on both unripe and ripe fruits [89]. Fruit availability throughout the field season due to production of day-neutral strawberry cultivars and dry and warm climate favors the development of multiple generations of *N. bilobata* [95]. Although there are some broad-spectrum insecticides available for management of *N. bilobata*, intercropping high-tunnel strawberry with garlic or undercropping with Chinese chives can suppress *N. bilobata* populations by 64% and 47%, respectively [96]. Traditional plant breeding resulting in strawberry fruits having seeds recessed into the fruit may provide protection against these achene feeders because of limited accessibility of the seeds [95].

### 2.8. Lepidopteran Pests

The major lepidopteran pests of strawberry include *Spodoptera frugiperda* (J.E. Smith), *Spodoptera eridania* Cramer, *Spodoptera exigua* (Hübner), *Agrotis ipsilon* (Hufnagel) (Lepidoptera: Noctuidae), *Duponchelia fovealis* Zeller (Lepidoptera: Crambidae), *Ancylis comptana* (Frölich), and *Acleris comariana* (Lienig and Zeller) (Lepidoptera: Tortricidae) [26,97,98,99]. The armyworm and cutworm pests may infest strawberry to feed on young foliage. *S. exigua* mostly damages strawberries planted in summer and fall. The immatures of *S. exigua* initially feed on strawberry foliage and eventually kill the plants by attacking the crown. Monitoring of *S. exigua* can be achieved through deploying pheromone traps [97]. Management can be achieved through cultural practices and biological control agents such as *Hyposoter exiguae* (Viereck) or application of commercial formulations of *Bacillus thuringiensis* [26,71]. 

In fact, in the case of an invasive pest, synthetic pesticides may not even be registered for use in the invasive range, in which case microbial insecticides may play an important role in the IPM of such pests. For example, [6] reported that *D. fovealis*, which is a recently invasive strawberry pest in Brazil, could not be managed with synthetic chemical products due to lack of product registration. As an alternative, commercial products based on the entomopathogenic fungus *B.* *bassiana* were tested and found to be compatible for the IPM of *D. fovealis* along with biological control agents such as the predatory stink bug, *Podisus nigrispinus* Dallas (Hemiptera: Pentatomidae) and the multicolored Asian lady beetle, *Harmonia axyridis* Pallas (Coleoptera: Coccinellidae)*. A. comariana* (Lienig and Zeller) (Lepidoptera: Tortricidae) is a common lepidopteran pest that attacks strawberry crops. A three-year study conducted in Denmark found that strawberry cropping practices had a significant impact on *A. comariana* management. *A. comariana* was more dominant on conventional farms than on organic farms and also the infestation was significantly higher in older fields compared to newer fields [99]. 

## 3. Conclusions

Augmentative biological control has emerged as an effective tool to manage several strawberry pests such as thrips, mites, plant bugs, weevils, spotted wing drosophila, aphids, whiteflies, and various lepidopteran pests (Table 1). However, conservation biological control tactics are yet to be utilized for strawberry pest management widely. The lack of adoption of conservation biological control can be attributed to the limited field research and cost-benefit analysis available. Since a successful conservation biological control program will require habitat manipulation to provide a physiological and behavioral advantage to natural enemies, future research programs need to be focused on these aspects. Laboratory research findings need to be expanded to large-scale field studies and supported with information regarding cost to promote adoption of this technology.

With the growing demand for organic strawberry across the globe, biological control has the potential to provide a sustainable pest management solution. We have a wealth of baseline information regarding potential of different species of flowering plants to function as insectary plants around strawberry fields. For example, pollen from strawberry, *Urtica urens*, *Lamium amplexicaule*, *Convolvulus arvensis*, *Sonchus oleraceous*, and *Galega officinalis* supports the development of predatory mite, *N. californicus*, to adulthood but does not support its reproduction [100]. For development and reproduction, *N. californicus* need to be supplemented with prey items such as *T. urticae* and thrips. This information can assist in the adoption of habitat manipulation programs that can sustain the development and reproduction of natural enemies to minimize chances of pest resurgence in strawberry fields. 

## Figures and Tables

**Table 1:** Summary table of pest management techniques for strawberry pests.

Strawberry Pest	Management	References
Thrips *Scirtothrips dorsalis* Hood *Frankliniella occidentalis* Pergande	Biological Control Predators: *Neoseiulus cucumeris* Oudemans, *Stratiolaelaps scimitus* (Womersley), *Amblyseius swirskii* Athias-Henriot, *Orius* spp., *Thripinema* spp. Cultural Control Eliminating the alternative host or weeds Chemical Control spinetoram, cyantraniliprole, flupyradifurone, imidacloprid + lambda, cyhalothrin, bifenthrin, neem extract	[18,19,20] [16] [10,21] [22]
Mites *Tetranychus urticae* Koch *Phytonemus pallidus* Banks	Physical Control Steam treatment at 48 °C for 2.7 h for adults and 1.9 h for eggs. Biological Control Predators: *Phytoseiulus persimilis* Athias-Henriot. *Neoseiulus californicus* McGregor Biopesticides: *Metarhizium anisopliae* strain F52, *Isaria fumosoroseus* Apopka strain 97, heat-killed *Burkholderia rinojensis* strain A396, heat-killed *Chromobacterium subtsugae* strain PRAA4-1 Chemical Control azadirachtin, paraffinic oil, potassium salts of fatty acids, unsulfonated residue of petroleum oil, neem oil Physical Control Steam treatment at 46 °C for 6.5 min Biological Control Predators: *Neoseiulus californicus* McGregor, *Neoseiulus cucumeris* Oudemans	[32] [26,33,100] [34,35,36]
Tarnished Plant Bug *Lygus lineolaris* (Palisot de Beauvois) *Lygus rugulipennis* Poppius	Physical Control Tractor-mounted suction machines Biological Control *Beauveria bassiana* (Balsamo-Crivelli) Vuillemin *Anaphes iole* Girault Cultural Control Trap crop, *Medicago sativa* L. Chemical Control hexyl butyrate + phenylacetaldehyde for Push-Pull Strategy flupyradifurone, novaluron, flonicamid, pyrethroids, organophosphates, neonicotinoids	[54,55,56] [51] [53] [41,58,59] [52] [49,50]
Beetles Sap Beetles *Haptoncus luteolus* (Erichson), *Lobiopa insularis* (Castelnau)*, Carpophilus fumatus* Boheman, *Stelidota geminate* (Say) Weevils *Otiorhynchus sulcatus* F., *O. ovatus* L., *O. singularis* L., *Otiorhynchus cribricollis* Gyllenhal, *Pantomorus cervinus* Boheman, *Nemocestes incomptus* Horn, *Barypeithes pellucidus* Boheman, *Sciopithes obscurus* L. *Anthonomus rubi* Herbst *Popillia japonica* Newman, *Anomala orientalis* Waterhouse, *Amphimallon majalis* Razoumowsky, *Maladera castanea* Arrow	Host Plant Resistance Cultivars with ripe fruits not touching the ground Cultural Control Field sanitation by removal of ripe fruits Chemical Control Pyrethroids Limiting fungicide application by using environmental- model-based spray program. Biological Control Entomopathogenic nematode, *Steinernema kraussei* (Steiner, 1923) Host Plant Resistance Cultivar “Symphony” showed lower feeding and oviposition. Cultural Control Strawberry bed sanitation by removal of weeds and grass. Chemical Control Pyrethroids Physical Control Yellow sticky card combined with synthetic attractants for adult monitoring and mass trapping. Periodic removal of severed buds or adults. Biological Control Braconidae *Pteromalus swederus* Cultural Control Crop rotation with “Saia” oats, *Avena strigosa* Schreb and “Garry” oats, *Avena sativa* L. Chemical Control Neem Oil Insecticidal Soaps	[63] [62] [62] [67] [68,71] [76] [77] [74] [71,78]
Spotted Wing Drosophila *Drosophila suzukii* Matsumura	Physical control Utilizing ultraviolet light; blue-green light spectrum (430–565 nm) Biological control *Orius insidiosus* (Say) along with *Heterorhabditis bacteriophora* Poinar	[83] [85]
Aphids and Whiteflies *Aphid* spp. *Trialeurodes vaporariorum Westwood* *Bemisia tabaci* Gennadius	Biological Control *Aphelinus varipes* (Foerster) *Aphidius colemani* Viereck Syrphid flies Green lacewings Lady beetles Physical Control Irradiation of boxed fresh fruits with gamma rays (100 Gy) Biological Control *Isaria fumosorosea* (Wize) Syrphid flies Green lacewings Lady beetles	[88] [89] [90]
Seed Bug *Neopamera bilobata* Say	Cultural Control Intercropping with garlic or undercropping with Chinese chives Host plant resistance Traditional plant breeding for varieties with seeds recessed into the fruit	[96] [95]
Lepidopteran Pests *Spodoptera exigua* (Hübner) *Duponchelia fovealis* Zeller *Acleris comariana* (Lienig and Zeller)	Physical control Deploying pheromone traps Biological control Predator: *Hyposoter exiguae* (Viereck) Biopesticide: Commercial formulations of *Bacillus thuringiensis* Biological control *Beauveria bassiana* (Balsamo-Crivelli) Vuillemin along with *Podisus nigrispinus* Dallas and *Harmonia axyridis* Pallas Cultural Control Improve strawberry cropping practices as less pest pressure experienced in organic strawberry and newer fields compared to conventional strawberry and older fields	[26] [71,97] [6] [99]

## References

[1] Bi J.L., Niu Z.M., Yu L., Toscano N.C. (2016). Resistance status of the carmine spider mite, *Tetranychus cinnabarinus* and the twospotted spider mite, *Tetranychus urticae* to selected acaricides on strawberries. Insect Sci..

[2] Tuovinen T. Risk of invasive arthropod pests related to climate change in the northernmost small fruit production area in EU. Proceedings of the Workshop on Berry Production in Changing Climate Conditions Cultivation Systems.

[3] Andreazza F., Haddi K., Oliveira E.E., Ferreira J.A.M. (2016). *Drosophila suzukii* (Diptera: Drosophilidae) arrives at Minas Gerais State, a main strawberry production region in Brazil. Fla. Entomol..

[4] Lue C.H., Mottern J.L., Walsh G.C., Buffington M.L. (2017). New record for the invasive spotted wing drosophila, *Drosophila suzukii* (Matsumura, 1931) (Diptera: Drosophilidae) in Anillaco, western Argentina. Proc. Entomol. Soc. Wash..

[5] Panthi B.R., Renkema J.M., Lahiri S., Liburd O.E. (2021). The short-range movement of *Scirtothrips dorsalis* (Thysanoptera: Thripidae) and rate of spread of feeding injury among strawberry plants. Environ. Entomol..

[6] Araujo E.S., Benatto A., Rizzato F.B., Poltronieri A.S., Poitevin C.G., Zawadneak M.A.C., Pimentel I.C. (2020). Combining biocontrol agents with different mechanisms of action to control *Duponchelia fovealis*, an invasive pest in South America. Crop Prot..

[7] FAO (2019). FAOSTAT Agricultural Statistics Database. http://www.fao.org/faostat/en/#data/QC/visualize.

[8] Strzyzewski I.L., Funderburk J.E., Renkema J.M., Smith H.A. (2021). Characterization of *Frankliniella occidentalis* and *Frankliniella bispinosa* (Thysanoptera: Thripidae) injury to strawberry. J. Econ. Entomol..

[9] Morse J.G., Hoddle M.S. (2006). Invasion biology of thrips. Annu. Rev. Entomol..

[10] Abdelmaksoud E.M., Elrefai S.A., Mahmoud K.W., Mohammed S.M. (2020). The effectiveness of some pesticides in the control of thrips and red spider mites on strawberry plants. Arab. Univ. J. Agric. Sci..

[11] Nielsen H., Sigsgaard L., Kobro S., Jensen N.L., Jacobsen S.K. (2021). Species composition of thrips (Thysanoptera: Thripidae) in strawberry high tunnels in Denmark. Insects.

[12] Renkema J.M., Evans B., Devkota S. (2018). Management of flower thrips in Florida strawberries with *Steinernema feltiae* (Rhabditida: Steinernematidae) and the insecticide sulfoxaflor. Fla. Entomol..

[13] Gremo F., Bogetti C., Scarpelli F. (1997). The thrips damaging to strawberry. L’Inf. Agrar..

[14] Buxton J.H., Easterbrook M.A. (1998). Thrips as a probable cause of severe fruit distortion in late-season strawberries. Plant Pathol..

[15] Steiner M.Y., Goodwin S. (2005). Management of thrips (Thysanoptera: Thripidae) in Australian strawberry crops: Within-plant distribution characteristics and action thresholds. Aust. J. Entomol..

[16] Kumar V., Kakkar G., Seal D.R., McKenzie C.L., Colee J., Osborne L.S. (2014). Temporal and spatial distribution of an invasive thrips species *Scirtothrips dorsalis* (Thysanoptera: Thripidae). Crop Prot..

[17] Hansen E.A., Funderburk J.E., Reitz S.R., Ramachandran S., Eger J.E., McAuslane H. (2003). Within-plant distribution of *Frankliniella* species (Thysanoptera: Thripidae) and *Orius insidiosus* (Heteroptera: Anthocoridae) in field pepper. Environ. Entomol..

[18] Kumar V., Kakkar G., McKenzie C.L., Seal D.R., Osborne L.S., Soloneski S., Larramendy M. (2013). An overview of chilli thrips, Scirtothrips dorsalis (Thysanoptera: Thripidae) biology, distribution and management. Weed and Pest Control-Conventional and New Challenges.

[19] Sampson C., Bennison J., Kirk W.D. (2001). Overwintering of the western flower thrips in outdoor strawberry crops. J. Pest Sci..

[20] Arthurs S., McKenzie C.L., Chen J., Dogramaci M., Brennan M., Houben K., Osborne L. (2009). Evaluation of *Neoseiulus cucumeris* and *Amblyseius swirskii* (Acari: Phytoseiidae) as biological control agents of chilli thrips, *Scirtothrips dorsalis* (Thysanoptera: Thripidae) on pepper. Biol. Control.

[21] Lahiri S., Panthi B. (2020). Insecticide efficacy for chilli thrips management in Strawberry, 2019. Arthropod Manag. Tests.

[22] Monteon-Ojeda A., Damian-Nava A., Hernandez-Castro E., Cruz-Lagunas B., Romero-Rosales T., San J.L.J., Ibarra-Cortes K.H. (2021). Biorational and conventional insecticides efficacy to control thrips (*Frankliniella occidentalis* Perg.) on strawberries (*Fragaria X ananassa* Duch.) at Morelos state, Mexico. AGRO Product..

[23] Zhao G., Liu W., Brown J.M., Knowles C.O. (1995). Insecticide resistance in field and laboratory strains of western flower thrips (Thysanoptera: Thripidae). J. Econ. Entomol..

[24] Jensen S.E. (2000). Insecticide resistance in the western flower thrips, *Frankliniella occidentalis*. Integr. Pest Manag. Rev..

[25] Renkema J.M., LeFors J.A., Johnson D.T. (2017). First report of broad mite (Acari: Tarsonemidae) on commercial strawberry in Florida. Fla. Entomol..

[26] Liburd O., Rhodes E., Asao T., Asaduzzaman M. (2019). Management of strawberry insect and mite pests in greenhouse and field crops. Strawberry-Pre-and Post-Harvest Management Techniques for Higher Fruit Quality.

[27] Easterbrook M.A., Fitzgerald J.D., Solomon M. (2001). Biological control of strawberry tarsonemid mite *Phytonemus pallidus* and two-spotted spider mite *Tetranychus urticae* on strawberry in the UK using species of *Neoseiulus* (Amblyseius) (Acari: Phytoseiidae). Exp. Appl. Acarol..

[28] Vacante V. (2016). The Handbook of Mites of Economic plants: Identification, Bio-Ecology and Control.

[29] Sato M.E., Da Silva M.Z., De Souza Filho M.F., Matioli A.L., Raga A. (2007). Management of *Tetranychus urticae* (Acari: Tetranychidae) in strawberry fields with *Neoseiulus californicus* (Acari: Phytoseiidae) and acaricides. Exp. Appl. Acarol..

[30] Bensoussan N., Zhurov V., Yamakawa S., O’Neil C.H., Suzuki T., Grbić M., Grbić V. (2018). The digestive system of the two-spotted spider mite, *Tetranychus urticae* Koch, in the context of the mite-plant interaction. Front. Plant Sci..

[31] Tuan S.J., Lin Y.H., Yang C.M., Atlihan R., Saska P., Chi H. (2016). Survival and reproductive strategies in two-spotted spider mites: Demographic analysis of arrhenotokous parthenogenesis of *Tetranychus urticae* (Acari: Tetranychidae). J. Econ. Entomol..

[32] Renkema J., Dubon F., Peres N., Evans B. (2020). Twospotted spider mites (*Tetranychus urticae*) on strawberry (*Fragaria × ananassa*) transplants, and the potential to eliminate them with steam treatment. Int. J. Fruit Sci..

[33] Howell A.D., Daugovish O. (2013). Biological control of *Eotetranychus lewisi* and *Tetranychus urticae* (Acari: Tetranychidae) on strawberry by four phytoseiids (Acari: Phytoseiidae). J. Econ. Entomol..

[34] Wang Z., Cang T., Wu S., Wang X., Qi P., Wang X., Zhao X. (2018). Screening for suitable chemical acaricides against two-spotted spider mites, *Tetranychus urticae*, on greenhouse strawberries in China. Ecotoxicol. Environ. Saf..

[35] Bernardi D., Botton M., da Cunha U.S., Bernardi O., Malausa T., Garcia M.S., Nava D.E. (2013). Effects of azadirachtin on *Tetranychus urticae* (Acari: Tetranychidae) and its compatibility with predatory mites (Acari: Phytoseiidae) on strawberry. Pest Manag. Sci..

[36] Whitaker V.M., Boyd N.S., Peres N.A., Desaeger J., Lahiri S., Dittmar P.J., Freeman J.H., Paret M.L., Smith H.A. (2020). Chapter 16. Strawberry Production. Vegetable Production Handbook of Florida.

[37] Croft B.A., Pratt P.D., Koskela G., Kaufman D. (1998). Predation, reproduction, and impact of phytoseiid mites (Acari: Phytoseiidae) on cyclamen mite (Acari: Tarsonemidae) on strawberry. J. Econ. Entomol..

[38] Fountain M.T., Harris A.L., Cross J.V. (2010). The use of surfactants to enhance acaricide control of *Phytonemus pallidus* (Acari: Tarsonemidae) in strawberry. Crop Prot..

[39] Patenaude S., Tellier S., Fournier V. (2020). Cyclamen mite (Acari: Tarsonemidae) monitoring in eastern Canada strawberry (Rosaceae) fields and its potential control by the predatory mite *Neoseiulus cucumeris* (Acari: Phytoseiidae). Can. Entomol..

[40] Hellqvist S. (2002). Heat tolerance of strawberry tarsonemid mite *Phytonemus pallidus*. Ann. Appl. Biol..

[41] Dumont F., Provost C. (2019). Combining the use of trap crops and insecticide sprays to control the tarnished plant bug (Hemiptera: Miridae) in strawberry (Rosaceae) fields. Can. Entomol..

[42] Schwartz M.D., Foottit R.G. (1998). Revision of the nearctic species of the genus *Lygus* Hahn, with a review of the palearctic species (Heteroptera: Miridae). Ann. Entomol. Soc..

[43] Udayagiri S., Welter S.C. (2000). Escape of *Lygus hesperus* (Heteroptera: Miridae) eggs from parasitism by *Anaphes iole* (Hymenoptera: Mymaridae) in strawberries plant structure effects. Biol. Control.

[44] Allen W.W., Gaede S.E. (1963). The relationship of lygus bugs and thrips to fruit deformity in strawberry. J. Econ. Entomol..

[45] Zalom F.G., Pickel C., Welch N.C., Bostanian N.J., Wilson L.T., Dennehy T.J. (1990). Recent trends in strawberry arthropod management for coastal areas of the western United States. Monitoring and Integrated Management of Arthropod Pests of Small Fruit Crops.

[46] Zalom F.G., Pickel C., Walsh D.B., Welch N.C. (1993). Sampling for *Lygus hesperus* (Hemiptera: Miridae) in strawberries. J. Econ. Entomol..

[47] Bolda M.A., Daugovish O., Fennimore S.A., Koike S.T., Larson K.D., Marcum D.B., Zalom F.G. (2008). Integrated Pest Management for Strawberries.

[48] Zalom F., Bi J., Thompson P. (2011). Managing Lygus Bugs in Strawberries. https://www.calstrawberry.com/Portals/2/Reports/ResearchReports/ProductionGuidelines/English/ManagingLygusBugsinStrawberries-2011.pdfver=2018-01-12-155446-573.

[49] Zalom F.G., Bolda M.P., Dara S.K., Joseph S.V. (2018). Strawberry Pest Management Guidelines. Lygus Bugs (Western Tarnished Plant Bug). https://www2.ipm.ucanr.edu/agriculture/strawberry/Lygus-Bug/.

[50] Fitzgerald J., Jay C. (2011). Chemical control of the European tarnished plant bug, *Lygus rugulipennis*, on strawberry in the UK. Crop Prot..

[51] Dara S. (2016). Managing strawberry pests with chemical pesticides and non-chemical alternatives. Int. J. Fruit Sci..

[52] Fountain M.T., Deakin G., Farman D., Hall D., Jay C., Shaw B., Walker A. (2021). An effective ‘push–pull’control strategy for European tarnished plant bug, *Lygus rugulipennis* (Heteroptera: Miridae), in strawberry using synthetic semiochemicals. Pest Manag. Sci..

[53] Norton A.P., Welter S.C., Flexner J., Jackson C.G., Debolt J.W., Pickel C. (1992). Parasitism of *Lygus hesperus* (Miridae) by *Anaphes iole* (Mymaridae) and *Leiophron uniformis* (Braconidae) in California strawberry. Biol. Control.

[54] Vincent C., Lachance P. (1993). Evaluation of a tractor-propelled vacuum device for management of tarnished plant bug (Heteroptera: Miridae) populations in strawberry plantations. Environ. Entomol..

[55] Pickel C., Zalom F.G., Walsh D.B., Welch N.C. (1994). Efficacy of vacuum machines for *Lygus hesperus* (Hemiptera: Miridae) control in coastal California strawberries. J. Econ. Entomol..

[56] Joseph S.V., Bolda M. (2018). Evaluating the potential utility of an electrostatic sprayer and a tractor-mounted vacuum machine for *Lygus hesperus* (Hemiptera: Miridae) management in California’s coastal strawberry. Crop Prot..

[57] Swezey S.L., Nieto D.J., Bryer J.A. (2007). Control of western tarnished plant bug *Lygus hesperus* Knight (Hemiptera: Miridae) in California organic strawberries using alfalfa trap crop and tractor-mounted vacuums. Environ. Entomol..

[58] Swezey S.L., Nieto D.J., Pickett C.H., Hagler J.R., Bryer J.A., Machtley S.A. (2014). Spatial density and movement of *Lygus* spp. parasitoid *Peristenus relictus* (Hymenoptera: Braconidae) in organic strawberries with alfalfa trap crops. Environ. Entomol..

[59] Hagler J.R., Nieto D.J., Machtley S.A., Swezey S.L. (2020). Predator demographics and dispersal in alfalfa trap-cropped strawberry. Entomol. Exp. Appl..

[60] Potter M.A., Price J.F., Habeck D.H., Schuster D.J., McCord Jr. E. (2013). A survey of sap beetles (Coleoptera: Nitidulidae) in strawberry fields in west central Florida. Fla. Entomol..

[61] Fornari R.A., Junior R.M., Bernardi D., Botton M., Pastori P.L. (2013). Evaluation of damage, food attractants and population dynamics of strawberry sap beetle. Hortic. Bras..

[62] Loughner R.L., Loeb G.M., Schloemann S., Demchak K. (2008). Evaluation of cultural practices for potential to control strawberry sap beetle (Coleoptera: Nitidulidae). J. Econ. Entomol..

[63] de Souza M.T., de Souza M.T., Rizzato F.B., Zawadneak M.A.C., Cuquel F.L. (2019). Feeding of *Lobiopa insularis* (Coleoptera: Nitidulidae) on strawberries. Crop Prot..

[64] Fornazier M.J., Carmo C.A.S.D.O., Teixeira C.P., Pereira E.B. (1986). Finding of the strawberry borer *Lobiopa insularis* in the State of Espirito Santo. EMCAPA Comun. Técnico.

[65] Salles L.A.B., Williams R.N. (1986). Broca-Do-Morango (Lobiopa insularis).

[66] Greco N., Cluigt N., Cline A., Liljesthrom G. (2017). Life history traits and life table analysis of *Lobiopa insularis* (Coleoptera: Nitidulidae) fed on strawberry. PLoS ONE.

[67] Swett C.L., Butler B.B., Peres N.A., Koivunen E.E., Hellman E.M., Beaulieu J.R. (2020). Using model-based fungicide programing to effectively control botrytis and anthracnose fruit rots in Mid-Atlantic strawberry fields and co-manage strawberry sap beetle (*Stelidota geminate*). Crop Prot..

[68] Labuschagne L., Wainwright H., Jennings D.L. (1997). Evidence of variation in susceptibility to vine weevil (*Otiorhynchus sulcatus*) in strawberry cultivars. Acta Hortic..

[69] Downes W. (1920). The strawberry root weevil in British Columbia. The 50th Annual Report of the Entomological Society of Ontario.

[70] Bomford M.K., Vernon R.S. (2005). Root weevil (Coleoptera: Curculionidae) and ground beetle (Coleoptera: Carabidae) immigration into strawberry plots protected by fence or portable trench barriers. Environ. Entomol..

[71] Reddy P.P. (2016). Strawberry. Sustainable Crop Protection under Protected Cultivation.

[72] Krauß A., Steen C., Zebitz C.P.W. Phenology of the strawberry blossom weevil and damage in strawberries. Proceedings of the 16th International Conference on Organic Fruit-Growing. Stuttgart-Hohenheim.

[73] Innocenzi P.J., Hall D.R., Cross J.V. (2001). Components of male aggregation pheromone of strawberry blossom weevil, *Anthonomus rubi* Herbst (Coleoptera: Curculionidae). J. Chem. Ecol..

[74] Franklin M.T., Hueppelsheuser T.K., Abram P.K., Bouchard P., Anderson R.S., Gibson G.A. (2021). The Eurasian strawberry blossom weevil, *Anthonomus rubi* (Herbst, 1795), is established in North America. Can. Entomol..

[75] Aasen S.S., Trandem N. (2006). Strawberry blossom weevil *Anthonomus rubi* Herbst (Col.: Curculionidae): Relationships between bud damage, weevil density, insecticide use, and yield. J. Pest Sci..

[76] Tonina L., Zanettin G., Miorelli P., Puppato S., Cuthbertson A.G., Grassi A. (2021). *Anthonomus rubi* on strawberry fruit: Its biology, ecology, damage, and control from an IPM perspective. Insects.

[77] Cross J.V., Hesketh H., Jay C.N., Hall D.R., Innocenzi P.J., Farman D.I., Burgess C.M. (2006). Exploiting the aggregation pheromone of strawberry blossom weevil *Anthonomus rubi* Herbst (Coleoptera: Curculionidae): Part 1. Development of lure and trap. Crop Prot..

[78] LaMondia J.A., Elmer W.H., Mervosh T.L., Cowles R.S. (2002). Integrated management of strawberry pests by rotation and intercropping. Crop Prot..

[79] Atallah J., Teixeira L., Salazar R., Zaragoza G., Kopp A. (2014). The making of a pest: The evolution of a fruit-penetrating ovipositor in *Drosophila suzukii* and related species. Proc. R. Soc. B.

[80] Boughdad A., Haddi K., El Bouazzati A., Nassiri A., Tahiri A., El Anbri C., Eddaya T., Zaid A., Biondi A. (2021). First record of the invasive spotted wing Drosophila infesting berry crops in Africa. J. Pest Sci..

[81] Bolda M.P., Goodhue R.E., Zalom F.G. (2010). Spotted wing drosophila: Potential economic impact of a newly established pest. Berkeley CA Univ. Calif..

[82] Zhu E.Y., Guntur A.R., He R., Stern U., Yang C.H. (2014). Egg-laying demand induces aversion of UV light in Drosophila females. Curr. Biol..

[83] Fountain M.T., Badiee A., Hemer S., Delgado A., Mangan M., Dowding C., Davis F., Pearson A. (2020). The use of light spectrum blocking films to reduce populations of *Drosophila suzukii* Matsumura in fruit crops. Sci. Rep..

[84] Gong X., Bräcker L., Bölke N., Plata C., Zeitlmayr S., Metzler D., Olbricht K., Gompel N., Parniske M. (2016). Strawberry accessions with reduced *Drosophila suzukii* emergence from fruits. Front. Plant Sci..

[85] Renkema J.M., Cuthbertson A.G. (2018). Impact of multiple natural enemies on immature *Drosophila suzukii* in strawberries and blueberries. BioControl.

[86] Ng J.C., Perry K.L. (2004). Transmission of plant viruses by aphid vectors. Mol. Plant Pathol..

[87] Bonneau P., Brisson J.D., Tellier S., Fournier V. (2019). Flight phenology and trap selection for monitoring potential viral vector Aphididae and Aleyrodidae (Hemiptera) in strawberry (Rosaceae) fields of Quebec, Canada. Can. Entomol..

[88] Peña-Martinez R., Munoz-Viveros A.L., Vanegas-Rico J.M., Rodriguez D., Hernandez R.A.T. (2020). Presence and distribution of *Aphis ruborum* with parasitoid *Aphidius colemani* in Mexico. Southwest. Entomol..

[89] Cho S.R., Koo H.N., Shin S., Kim H.K., Park J.H., Yoon Y.S., Kim G.H. (2019). Gamma-ray irradiation control of whiteflies *Bemisia tabaci* (Hemiptera: Aleyrodidae) and *Trialeurodes vaporariorum* in the exportation of fresh strawberries. J. Econ. Entomol..

[90] Cuellar-Sandoval J.F., Ortega-Martínez L.D., Hernández-Velázquez V.M., Cuellar-Zometa J.F., Torres-García G., Barrales-Cureño H.J., Salazar-Magallón J.A. (2019). Agroecological basis for the design of biotechnological traps based on *Isaria fumosorosea* for the biological control of *Bemisia tabaci* in strawberry crops. Biocontrol Sci. Technol..

[91] Brooks A.N., Watson J.M.R., Mowry H. (1929). Strawberries in Florida: Culture, Disease, and Insects.

[92] Dellape P.M. (2005). Biodiversidad, Relationships Genetics and Biogeographic Aspects of Rhyparochromidae (Lygaeoidea: Heteroptera) with Special Reference to the Genus *Neopamera* Harrington 1980. Master’s Thesis.

[93] Kuhn T.M., Loeck A.E., Zawadneak M.A., Garcia S., Button M. (2014). Parameters biological and fertility life table of *Neopamera bilobata* (Hemiptera: Rhyparochromidae) in strawberry. Brasilia.

[94] Bernardi D., Andreazza F., Nava D.E., Baronio C.A., Botton M. (2015). Duplo Ataque. Cultivar HF.

[95] Talton H.R., Rhodes E.M., Chase C.A., Swisher M.E., Renkema J.M., Liburd O.E. (2020). Effect of cultural practices on *Neopamera bilobata* in relation to fruit injury and marketable yields in organic strawberries. Insects.

[96] Hata F.T., Ventura M.U., Bega V.L., Camacho I.M., Paula M.T. (2019). Chinese chives and garlic in intercropping in strawberry high tunnels for *Neopamera bilobata* Say (Hemiptera: Rhyparochromidae) control. Bull. Entomol. Res..

[97] Asano S., Nagaoka H., Wada Y., Miyamoto K. (2004). Influence of leaves on the biological activity of *Bacillus thuringiensis* formulation against the common cutworm, *Spodoptera litura*. Jpn. J. Appl. Entomol. Zool..

[98] Bortoli L.C., Bertin A., Efrom C.F.S., Botton M. (2012). Biology, fertility life table and effect of insecticides on *Spodoptera eridania* (Cramer) (Lepidoptera: Noctuidae) in strawberry and grape. Rev. Bras. Frutic. Jaboticabal..

[99] Sigsgaard L., Naulin C., Haukeland S., Kristensen K., Enkegaard A., Jensen N.L., Eilenberg J. (2014). The effects of strawberry cropping practices on the strawberry tortricid (Lepidoptera: Tortricidae), its natural enemies, and the presence of nematodes. J. Insect Sci..

[100] Ottaviano M.F.G., Cédola C.V., Sánchez N.E., Greco N.M. (2015). Conservation biological control in strawberry: Effect of different pollen on development, survival, and reproduction of *Neoseiulus californicus* (Acari: Phytoseiidae). Exp. Appl. Acarol..

